# The association between anxiety levels and postoperative pain following endodontic treatment: a prospective observational study

**DOI:** 10.1186/s12903-026-08715-7

**Published:** 2026-05-27

**Authors:** Hatice Sağlam, Hakan Arslan

**Affiliations:** İstanbul Medeniyet University Faculty of Dentistry Department of Endodontics, İstanbul, Türkiye

**Keywords:** Anxiety, Dental fear, Endodontics, Postoperative pain, Root canal treatment

## Abstract

**Background:**

The aim of this study is to investigate the association between preoperative anxiety and postoperative pain after endodontic treatment in vital symptomatic mandibular molar teeth.

**Methods:**

132 patients who came for endodontic treatment of symptomatic molars were asked to complete the ‘Modified Dental Anxiety Scale’ (MDAS) to assess preoperative anxiety. After the treatment, patients were provided with a pain assessment form and requested to record average daily pain intensity for seven days. Postoperative pain was assessed using a Visual Analog Scale (VAS). The completed forms were collected one week later. Data were analyzed using Mann-Whitney-U test, Chi-square test, one-way ANOVA, Kruskal-Wallis and multivariable linear regression analysis tests. The significance level was set at *p* < 0.05.

**Results:**

Analysis of postoperative pain values revealed a significant difference between groups in the first 3 days postoperatively (*p* < 0.05). Post-hoc pairwise comparisons performed for the first three postoperative days demonstrated a statistically significant difference in pain reduction, particularly between the moderate and high anxiety groups (*p* < 0.05). Regarding the relationship between sex and postoperative pain, a significant difference was found only on postoperative day 3 (*p*= 0.031). A statistically significant difference was observed among the groups regarding postoperative analgesic use (*p* < 0.001). Multivariable regression analysis indicated that anxiety was associated with postoperative pain after adjusting for potential confounders (*p* = 0.018).

**Conclusion:**

In conclusion dental anxiety may influence postoperative pain. Patients with higher dental anxiety tend to experience greater postoperative pain. Evaluating patients’ anxiety levels can contribute to more effective management of the treatment, and the implementation of anxiety-reducing strategies may help to lower the risk of postoperative pain.

**Supplementary Information:**

The online version contains supplementary material available at 10.1186/s12903-026-08715-7.

## Background

Anxiety is, at its core, a fear of the unknown [1]. Dental anxiety refers specifically to a patient’s response to the stress associated with dental treatment or to the anticipation of an unpleasant event during any dental procedure [2]. Dental anxiety is a significant factor that complicates the delivery of dental treatments. A substantial proportion of adults experience varying degrees of dental anxiety, and it is estimated that approximately 3–16% of adults suffer from dental phobia [3]. Dental anxiety may emerge during childhood or develop later in life and multiple factors can contribute to its onset, including lack of knowledge about dental procedures, cultural differences, limited awareness of oral health, misconceptions regarding dental treatments, negative or traumatic past experiences, as well as fear of dental instruments, needles, or blood [4, 5]. Moreover, worrying about the possibility of experiencing pain during a dental visit may also contribute to the development of dental anxiety [6, 7]. Being aware of these factors is crucial for understanding and addressing anxiety related to dental treatment [8]. Evaluating patients’ anxiety levels is essential for delivering high-quality care, minimizing treatment-related stress, and promoting optimal postoperative recovery [1, 9]. Adopting an appropriate approach to managing an anxious patient can contribute to the effective management of the treatment process for both the patient and the clinician. Several scales have been developed to measure the level of dental anxiety. Some of the scales applied to assess dental anxiety in adults include the Corah Dental Anxiety Scale (DAS), the Modified Dental Anxiety Scale (MDAS), the Dental Fear Survey (DFS), the Modified Dental Fear Survey (MDFS), and the Dental State Anxiety Scale (DSAS) [10]. The DAS, developed by Corah in 1969, was the first dental anxiety scale and is easy to use as it consists of only four questions [11]. However, it does not provide an assessment related to local anesthesia. In 1995, twenty-six years after the development of the DAS, the MDAS was introduced by Humphris and colleagues from the United Kingdom [10]. The MDAS is a modified version of Corah’s Dental Anxiety Scale, with an additional question regarding local anesthetic injection [12]. In this questionnaire, the response scores yield a range between 5 and 25 points, with higher scores indicating higher levels of anxiety. Endodontic treatments represent a branch of dentistry that is closely associated with pain. Endodontic procedures, such as root canal treatment, are often associated with fear and anxiety due to factors such as perceived pain, discomfort, and uncertainty regarding the outcome [13]. Among dental procedures, tooth extractions are reported to cause the highest level of anxiety, followed by root canal treatment [2]. Additionally, a study [14] demonstrated a statistically significant association between dental anxiety and a history of invasive dental procedures based on dental records. In the literature, studies have examined the relationship between dental anxiety and endodontic procedures. However, these studies have mostly focused on the association between anxiety and pain experienced during treatment. In a study on adult patients with reversible pulpitis, Dou et al. [15] found that pain experienced during endodontic treatment was strongly correlated with dental anxiety. Other studies have also reported a positive correlation between preoperative anxiety levels and pain experienced during endodontic treatment [15–17]. In contrast, evidence regarding the relationship between anxiety levels and postoperative pain in the field of endodontics remains limited. Most recently, Vitali et al. [18] investigated patient-related factors contributing to postoperative pain and suggested that dental anxiety is one of these factors. Another study published in 2024 [19] examined the impact of patient anxiety on the effectiveness of endodontic procedures. According to measurements taken at three different time points, postoperative pain was lower in patients with low anxiety levels compared to those with high anxiety levels. However, this study did not provide detailed information about the endodontic treatments performed, nor did it examine the relationship between sex and pain. The complex nature of postoperative pain has limited the frequency with which it has been investigated in endodontics.

To the best of our knowledge, no study has examined the impact of patients’ anxiety levels on postoperative pain in vital symptomatic mandibular molars using different parameters. Therefore, the aim of the present study was to evaluate postoperative pain following endodontic treatment of symptomatic vital mandibular molars over a one-week period and to explore its association with dental anxiety, as well as physiological and vital parameters.

## Materials and methods

### Study design and patient participation

The present study was explicitly designed as a prospective observational study to compare postoperative pain outcomes over a one-week period in patients requiring endodontic treatment with different levels of anxiety. Ethical approval for the study was obtained from Biruni University Clinical Research Ethics Committee (28.07.2022- Decision No: 2022/72 − 06) (Annex 1). All procedures involving participants were conducted in accordance with the Declaration of Helsinki. The findings of this study were reported in accordance with the Strengthening the Reporting of Observational Studies in Epidemiology (STROBE) statement to ensure the highest quality of reporting. Informed consent was obtained from all patients after they were provided with information about the aims and nature of the study (Annex 2). The study was carried out in Biruni University, Faculty of Dentistry, Department of Endodontics (İstanbul, Türkiye) between August 2022 and March 2023.

### Inclusion criteria

Inclusion criteria were as follows;

- Patients over 18 years of age.
- Symptomatic mandibular molars with requiring endodontic treatment.
- Vital teeth (positive response to thermal pulp testing, bleeding from pulp chamber and absence of apical radiolucency).

### Exclusion criteria

- Presence of any systemic disease requiring prophylaxis.
- Pregnancy.
- Chronic pain or use of analgesic/anti-inflammatory medication within the last 48 h.
- Presence of swelling or fistula.
- Teeth with periapical radiolucency.
- Severe periodontal problems or tooth mobility.
- Internal or external root resorption.
- Teeth that had previously undergone root canal treatment.

**Fig. 1:**
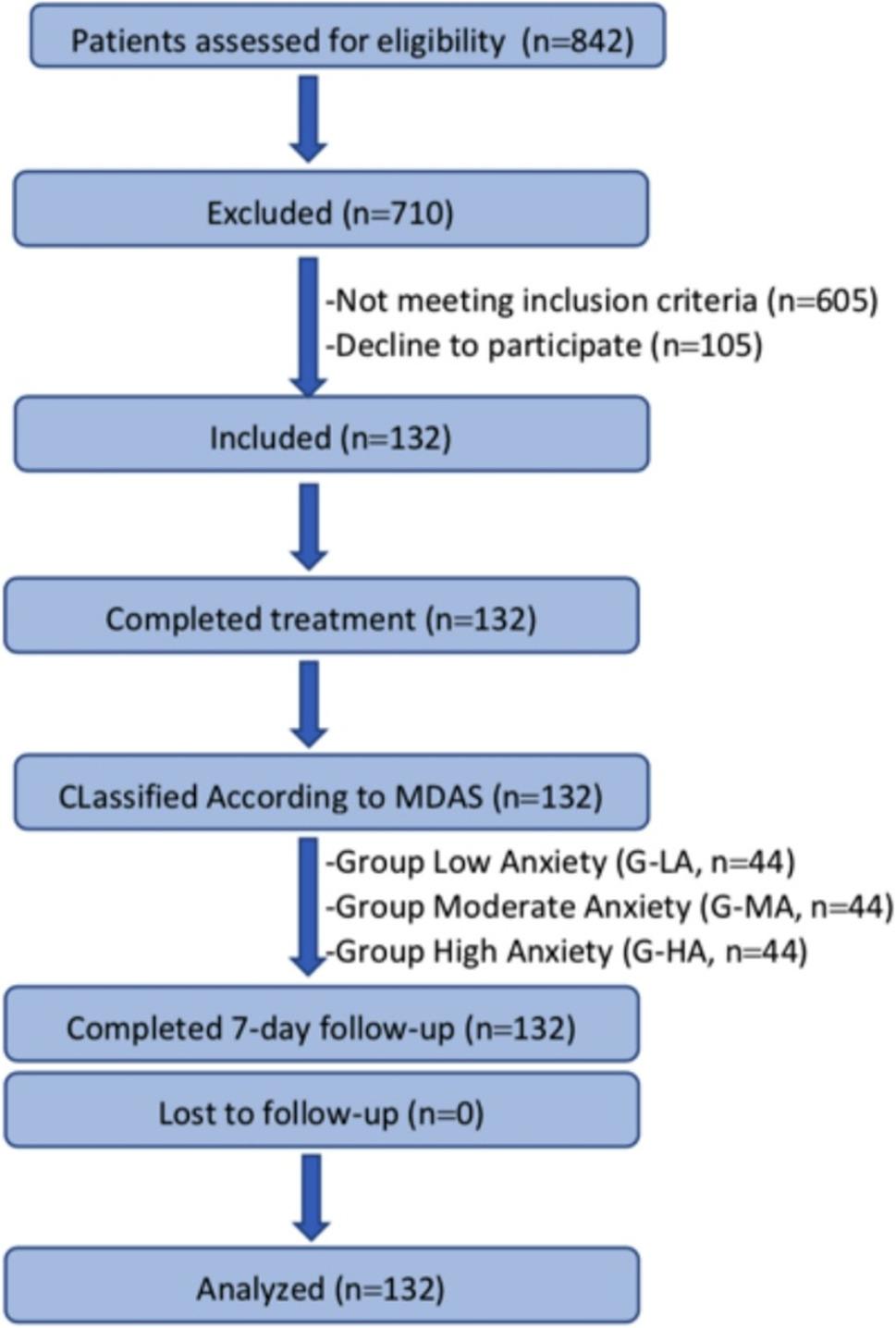
Flow diagram of participant recruitment, grouping, and follow-up in the observational study

A total of 842 patients who presented to the clinic were initially assessed for eligibility. Of these, 710 patients were excluded because they did not meet the inclusion criteria or declined to participate. A flow diagram summarizing participant recruitment, grouping, and follow-up is presented in Fig. 1. The study was conducted on patients who required endodontic treatment and voluntarily agreed to participate the study at the Department of Endodontics.

### Sample size calculation

An a priori power analysis was performed using G*Power 3.1 (Heinrich Heine University, Düsseldorf, Germany) for a one-way ANOVA with three groups. With an assumed effect size of f = 0.29, an α level of 0.05, and a desired power of 0.80, the required total sample size was calculated as 120 participants. To compensate for potential attrition, the final sample size was increased to 132 participants (44 per group).

### Experimental groups

**Fig. 2:**
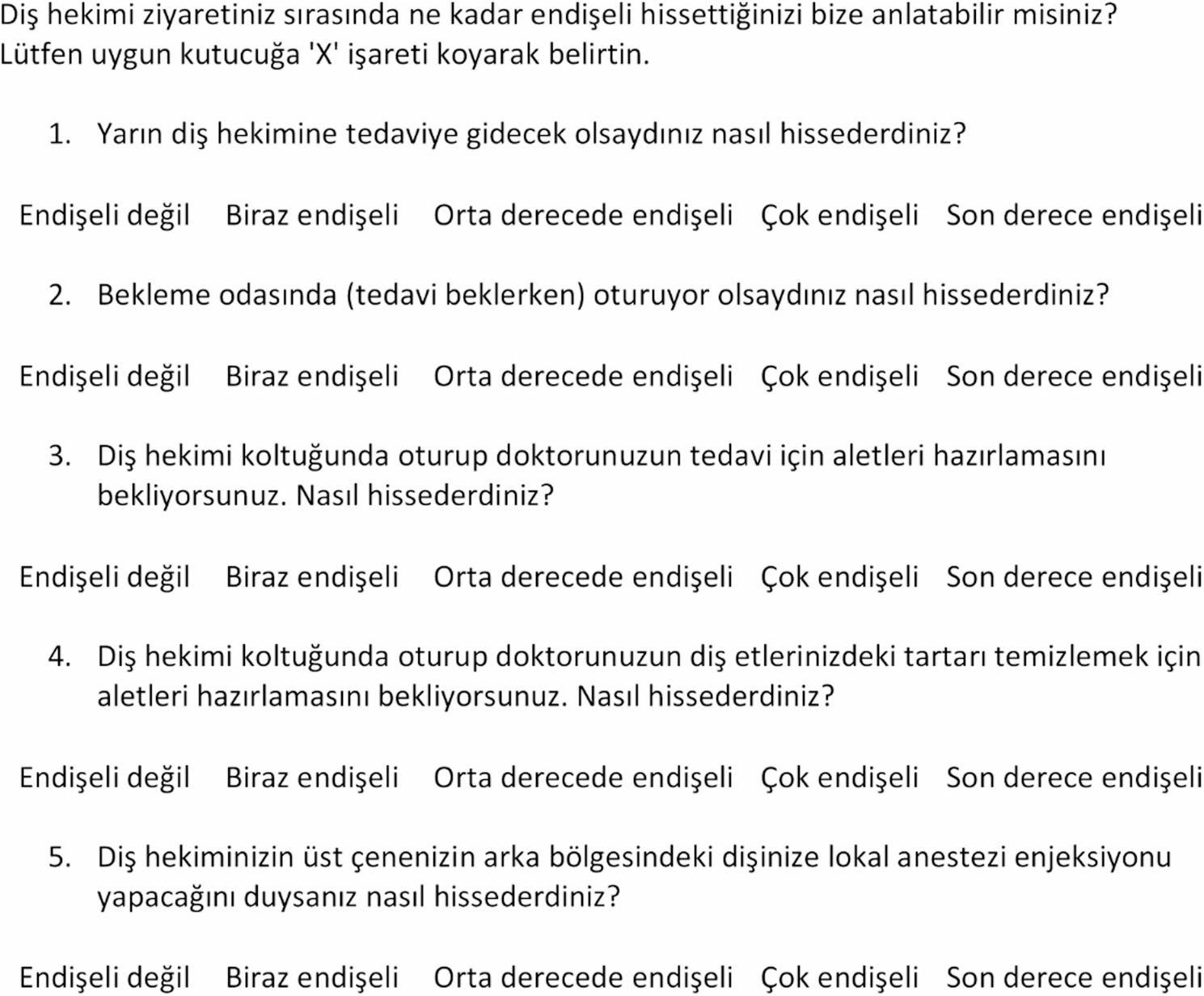
Turkish version of the Modified Dental Anxiety Scale (MDAS) used in the study

A total of 132 patients with varying levels of anxiety were included in the study. During the initial examination, the following demographic and clinical data were recorded: age, sex, pulse rate, body temperature, oxygen saturation, blood pressure (systolic and diastolic), preoperative spontaneous pain level and preoperative percussion sensitivity (recorded as percentages). Patients were recruited consecutively, and the Modified Dental Anxiety Scale (MDAS) was administered prior to treatment to assess their anxiety levels (Fig. 2). A validated Turkish version of the MDAS was used in the present study [20]. The questionnaire consisted of five items focusing on dental fear and anesthetic injections, presented with standardized response options. Each response was scored on a scale from 1 to 5: ‘not anxious’ = 1, ‘slightly anxious’ = 2, ‘fairly anxious’ = 3, ‘very anxious’ = 4, and ‘extremely anxious’ = 5, resulting in a total sum score of 5–25. Participants with MDAS scores ≥ 19 (19–25) were classified as having high dental anxiety according to established cut-off values [21]. For analytical purposes, the remaining participants were further categorized into low (5–9) and moderate (10–18) anxiety groups, consistent with previous study [22]. Based on the total scores obtained from the scale, patients were divided into three categories:

- Group Low Anxiety (G-LA): 5–9 points.
- Group Moderate Anxiety (G-MA): 10–18 points.
- Group High Anxiety: (G-HA): 19–25 points.

Following the questionnaire, all teeth underwent endodontic treatment using the same protocol, and all procedures were performed by the same operator. The operator was not aware of the patients’ anxiety scores during treatment. Anxiety scores were calculated for each patient immediately after treatment, and patients were divided into groups. Recruitment continued until each anxiety category reached the target sample size (*n* = 44), as determined by the power analysis.

### Root canal treatment protocol

Root canal treatment was performed in a single visit for all patients using a standardized protocol and no complications were experienced during the treatment. Local anesthesia was administered using standard techniques, and additional anesthesia was provided when required to achieve adequate pain control. Treatment procedures were initiated only after sufficient anesthesia was confirmed clinically. After administration of local anesthesia (1.8 mL of 2% lidocaine with 1:100,000 epinephrine, DFL; Taquara, RJ, Brazil), rubber dam isolation was achieved and an endodontic access cavity was prepared. The presence of root canals was confirmed with a #10 K-file (Mani Inc., Utsunomiya, Tochigi, Japan). Working length was determined using an electronic apex locator (Propex Pixi; Dentsply Maillefer, Ballaigues, Switzerland). After establishing a glide path, the mesial canals were prepared with VDW Rotate instruments to size 25.06, whereas the distal canal was prepared to size 40.06 if single or to size 25.06 if double (VDW, Munich, Germany). Irrigation of the root canals was performed using a 30-gauge side-vented irrigation needle positioned 3 mm short of the working length. For each canal, 4 mL of 5.25% NaOCl (Wizard, Istanbul, Turkey) was used, followed by a final irrigation with 3 mL of 17% EDTA (Werax, Izmir, Turkey) and 0.9% saline (Eczacıbaşı-Baxter, Istanbul, Turkey), applied alternately for 3 min. The root canals were dried with paper points (Pearl Endo; Beraydent, Ankara, Turkey). Root canal obturation was performed using AH Plus sealer (Dentsply Sirona, Germany) and gutta-percha cones (VDW, Munich, Germany) appropriate for the preparation with the lateral condensation technique. After sealing the pulp chamber with a flowable composite resin (GC Essentia Flow; GC Corp., Tokyo, Japan), the coronal restoration was completed using a nanohybrid composite resin (GC G-ænial Posterior A2; GC Corp., Tokyo, Japan).

### Pain assessment

Preoperative pain was assessed using a Visual Analog Scale (VAS; 0–10 cm, 0 = no pain, 10 = worst imaginable pain). After treatment, the patients were provided with a postoperative pain assessment form (Annex 3) and instructed to record their average daily pain intensity starting from the first postoperative day (24 h after treatment) and continuing for seven consecutive days. Postoperative pain was assessed using a VAS, similar to the evaluation of preoperative pain. Patients were also requested to report whether they used analgesics and to return to the clinic if pain persisted despite medication. Analgesic consumption was recorded as a binary variable (yes/no). The type, dosage, frequency, and duration of analgesic use were not standardized or recorded. Since patients may unintentionally modify their responses when reporting pain verbally to the clinician, self-completion of the pain form was used to minimize reporting and observer bias. The completed forms were collected at the one-week follow-up visit. The marked scores were subsequently converted to percentages based on their corresponding position on the scale, with the entire scale considered as 100 units. The VAS scores were converted to a 0–100 scale to express pain intensity as a percentage of the maximum possible pain, thereby improving interpretability and facilitating comparison across patients. This transformation is mathematically equivalent to the original 0–10 scale and does not influence the statistical outcomes. For example, if a patient marked a point corresponding to 70 units on a 0–100 scale, the pain level was recorded as 70%. Because patients’ perceptions were the primary outcome variables in the present study, all subjective data were collected in the absence of the clinician to avoid any potential influence of the clinician’s presence on patient perception.

### Statistical analyses

Statistical analyses were performed using IBM SPSS Statistics, version 22.0 (IBM Corp., Armonk, NY, USA). The normality of continuous variables was evaluated using the Shapiro–Wilk test prior to the comparison of demographic and preoperative values among the groups and appropriate parametric or non-parametric tests were selected based on the distribution of the data. Preoperative pain, preoperative percussion pain sensitivity (expressed as percentages), age, oxygen saturation, and systolic and diastolic blood pressure, which did not show a normal distribution, were analyzed using the Kruskal–Wallis test for comparisons among groups. Pulse rate and body temperature, which met the assumptions of normality, were analyzed using one-way ANOVA. When the Kruskal–Wallis test revealed a statistically significant difference, post-hoc pairwise comparisons were performed using the Mann–Whitney U test, and Bonferroni correction was applied to control for the increased risk of type I error due to multiple comparisons. Categorical variables were analyzed using the Chi-square test. To adjust for potential confounding factors, a multivariable linear regression analysis was performed with postoperative pain on day 1 as the dependent variable. Anxiety level was entered as a categorical variable (low anxiety as the reference group), and preoperative pain, sex, age, and analgesic intake were included as covariates. Regression coefficients (β) and corresponding p-values were calculated, and statistical significance was set at *p* < 0.05.

## Results

A total of 132 patients (63 males and 69 females) were included in the study and allocated into three groups based on their MDAS scores. Although patient replacement was planned in case of possible dropouts, no loss to follow-up occurred during the study period. Age, oxygen saturation, systolic blood pressure, diastolic blood pressure, and preoperative percussion sensitivity (expressed as percentages), which did not show a normal distribution, were analyzed using the Kruskal–Wallis test, and no statistically significant differences were found among the groups (*p* > 0.05). No statistically significant differences were found among the groups in terms of preoperative pain (*p* = 0.106), suggesting baseline comparability. Pulse rate and body temperature, which met the assumptions of normality, were analyzed using one-way ANOVA, and no significant differences were found among the anxiety groups (*p* = 0.415, *p*= 0.580). The preoperative and demographic data are presented in Table 1. Categorical variables were analyzed using the Chi-square test, and no significant difference was found among the groups in terms of sex distribution (*p* = 0.695). The sex distribution of the groups is shown in Table 2.

**Table 1:** Demographic and preoperative clinical variables according to anxiety groups

	G-LA (*n*=44)	G-MA (*n*=44)	G-HA (*n*=44)	p^∗^
Pulse	83.5 ± 11.6	86.2 ± 13.3	86.7 ± 12	0.415ᵃ
Body temperature (°C)	36.8 ± 0.5	36.8 ± 0.4	36.9 ± 0.4	0.580ᵃ
Preoperative pain (%)	0 (0–50)	22 (0–50)	20 (0–25)	0.106ᵇ
Age	29 (20.8–38)	29 (21–43.2)	32 (21.8–39.2)	0.906^b^
Saturation (%)	97 (96–97)	97 (95.75–98)	97 (96.75–98)	0.131^b^
SBP (mmHg)	115 (110–122.5)	120 (110–120)	120 (110–120)	0.920^b^
DBP (mmHg)	70 (60–80)	75 (70–80)	70 (70–80)	0.374^b^
Preoperative Percussion	0 (0–22.2)	20 (0–50)	25 (0–25)	0.403^b^
Sensitivity (%)				

*SBP* Systolic Blood Pressure, *DBP* Diastolic Blood Pressure, *G-LA* Group Low Anxiety, *G-MA* Group Moderate Anxiety, *G-HA* Group High Anxiety

^∗^< 0.05

ᵃ One-way ANOVA, ᵇ Kruskal–Wallis test, Data are presented as mean ± standard deviation for normally distributed variables and median (interquartile range, IQR) for non-normally distributed variables

**Table 2:** Sex distribution in term of groups

	Female (%)	Male (%)	*p* ^∗^
G-LA	21 (47.7%)	23(52.3%)	0.695
G-MA	25 (56.8%)	19(43.2%)	
G-HA	23 (52.3%)	21(47.7%)	

*G-LA* Group Low Anxiety, *G-MA* Group Moderate Anxiety, *G-HA* Group High Anxiety

^∗^< 0.05, Chi-Square test

In the analysis of postoperative pain values over the 7-day period according to anxiety groups, a statistically significant difference was found among the groups during the first three postoperative days with higher postoperative pain scores observed in G-HA compared to the other groups (*p* < 0.05). The postoperative pain values of the groups over the 7-day period are presented in Table 3. Regarding the relationship between sex and postoperative pain, a statistically significant difference was found only on postoperative day 3 (*p* = 0.031). The results of the postoperative pain analysis according to sex are presented in Table 4. Since the data were not normally distributed, changes in pain among the three anxiety groups were evaluated using the Kruskal–Wallis test. The analysis revealed statistically significant differences among the groups on postoperative days 1, 2, and 3 (*p* < 0.05), whereas no significant differences were observed on postoperative days 4 to 7 (*p* > 0.05). In the delta pain analysis (ΔPain: postoperative pain – preoperative pain), post-hoc pairwise comparisons performed for the first three postoperative days, which were found to be significant in the Kruskal–Wallis test, demonstrated a statistically significant difference in delta pain values particularly between G-MA and G-HA (*p* < 0.05). No significant differences were observed in the other group comparisons after Bonferroni correction (*p* > 0.05). Changes in pain scores among the anxiety groups are presented in Table 5. When postoperative analgesic consumption was evaluated, a statistically significant difference in analgesic consumption was observed among the groups, with higher use in G-HA compared to the other groups (*p* < 0.001). Information regarding analgesic consumption according to the groups is presented in Table 6. A multivariable linear regression analysis was conducted to evaluate the independent effect of anxiety on postoperative pain. When adjusted for preoperative pain, sex, age, and analgesic intake, the high anxiety group (G-HA) remained significantly associated with increased postoperative pain on day 1 (β = 16.78, 95% CI: 2.98–30.57, *p* = 0.018). The moderate anxiety group (G-MA) did not show a statistically significant difference compared to the low anxiety group (β = 5.38, *p* = 0.424). Preoperative pain (*p* = 0.425), sex (*p* = 0.170), and age (*p* = 0.289) were not significantly associated with postoperative pain, while analgesic intake showed a borderline association (β = 14.28, *p* = 0.086). The overall model was statistically significant (F = 2.965, *p* = 0.009), explaining 12.5% of the variance in postoperative pain (R² = 0.125; adjusted R² = 0.083). Information regarding regression analysis is presented in Table 7.

**Table 3:** Postoperative Pain Analysis According to Anxiety Groups (7 days)

	G-LA (*n*=44)	G-MA (*n*=44)	G-HA (*n*=44)	p^∗^
Post-op Day 1	20 (0–21)	7.5 (0–50)	35 (0–75)	0.006
Post-op Day 2	0 (0–20)	0 (0–50)	25 (0–50)	0.001
Post-op Day 3	0 (0–20)	0 (0–20)	22.5 (0–50)	0.022
Post-op Day 4	0 (0–5)	0 (0–5)	0 (0–50)	0.080
Post-op Day 5	0 (0–5)	0 (0–0)	0 (0–25)	0.169
Post-op Day 6	0 (0–0)	0 (0–0)	0 (0–21.2)	0.053
Post-op Day 7	0 (0–0)	0 (0–0)	0 (0–0)	0.278

Since the data did not follow a normal distribution, the values are given as median (IQR)

*G-LA* Group Low Anxiety, *G-MA* Group Moderate Anxiety, *G-HA* Group High Anxiety

^∗^< 0.05, Kruskal–Wallis test

**Table 4:** Postoperative pain analysis according to sex groups (7 days)

	Female (*n*=69)	Male (*n*=63)	p^∗^
Post-op Day 1	25 (0–50)	20 (0–25)	0.157
Post-op Day 2	20 (0–50)	0 (0–25)	0.264
Post-op Day 3	0 (0–50)	0 (0–20)	0.031
Post-op Day 4	0 (0–25)	0 (0–0)	0.075
Post-op Day 5	0 (0–20)	0 (0–0)	0.209
Post-op Day 6	0 (0–0)	0 (0–0)	0.229
Post-op Day 7	0 (0–0)	0 (0–0)	0.581*

Since the data did not follow a normal distribution, the values ​​are given as median (IQR)

^∗^< 0.05, Mann-Whitney U test

**Table 5:** ΔPain (Postoperative − Preoperative) Across Days 1–7 by Groups

	G-LA (*n*=44)	G-MA (*n*=44)	G-HA (*n*=44)	p^∗^
Day 1	0 (–7–20)^a^	0 (–25–25)^a^	25 (–2.5–50)^a^	0.021
Day 2	0 (–15–20)^ab^	0 (–25.5–0)^a^	7.5 (0–31.2)^b^	0.012
Day 3	0 (–24–20)^ab^	− 8.5 (–30–0)^a^	0 (–13.8–25)^b^	0.026
Day 4	0 (–24–0)	− 20 (–30–0)	0 (–25–21.2)	0.056
Day 5	0 (–24– 0)	− 20 (–35–0)	0 (–25–0)	0.093
Day 6	0 (–25–0)	− 20 (–50–0)	0 (–25–0)	0.076
Day 7	0 (–30–0)	− 21 (–50–0)	0 (–25–0)	0.052

Since the data did not follow a normal distribution, the values ​​are given as median (IQR)

Different superscript letters (a, b) indicate statistically significant differences between groups based on post-hoc pairwise comparisons using the Mann–Whitney U test with Bonferroni correction

*G-LA* Group Low Anxiety, *G-MA* Group Moderate Anxiety, *G-HA* Group High Anxiety

< 0.05, Kruskal–Wallis test

**Table 6:** Postoperative analgesic intake according to groups

	Analgesic Consumption, *n* (%)	*p* ^∗^
G-LA	2 (4.5%)ᵃ	< 0.001
G-MA	3 (6.8%)ᵃ	
G-HA	14 (31.8%)ᵇ	

Post-hoc pairwise comparisons were conducted using the Chi-square test with Bonferroni correction

Different superscript letters (a, b) indicate statistically significant differences between groups

*G-LA* Group Low Anxiety, *G-MA* Group Moderate Anxiety, *G-HA* Group High Anxiety

^∗^< 0.05, Chi-Square Test

**Table 7:** Multivariable linear regression analysis for postoperative pain (Postoperative Day 1)

Variable	β	CI (%95)	*p* ^∗^
G-MA (vs. G-LA)	5.38	-7.88 to 18.64	0.424
G-HA (vs. G-LA)	16.78	2.99 to 30.57	0.018
Preoperative pain	0.076	-0.11 to 0.27	0.425
Sex	-7.48	-18.20 to 3.24	0.170
Age	0.243	-0.21 to 0.69	0.289
Analgesic intake	14.28	-2.05 to 30.61	0.086

β: unstandardized regression coefficient (Model fit: R² = 0.125; adjusted R² = 0.083; F = 2.965; *p* = 0.009.)

*CI* Confidence interval, *G-LA* Group Low Anxiety (reference), *G-MA* Group Moderate Anxiety, *G-HA* Group High Anxiety

^∗^< 0.05

## Discussion

The present study comprehensively analyzed the effects of dental anxiety on postoperative pain. Symptomatic mandibular molars, representing a clinically significant and challenging group in endodontic procedures in terms of anesthesia and pain management, were selected to more precisely evaluate the relationship between anxiety and postoperative pain. Preoperative anxiety has been shown to have a significant effect on the modulation of postoperative pain in several studies [23–25]. A study published in 2018 [15] emphasized a strong positive correlation between dental anxiety and preoperative pain in patients with irreversible pulpitis. Wang et al. [26] reported a significant association between preoperative anxiety and postoperative pain in patients undergoing mandibular third molar extraction. Similarly, Gonçalvez et al. [27] demonstrated that preoperative anxiety influences both intraoperative discomfort and the memory of postoperative pain. In a study involving asymptomatic molars, Vitali et al. [18] identified an association between higher levels of dental anxiety and increased postoperative pain. The present study conducted on symptomatic molars found that higher preoperative dental anxiety was associated with greater early postoperative pain. Importantly, this association remained significant after adjusting for potential confounding variables, including preoperative pain, sex, age, and analgesic intake, suggesting that anxiety may act as an independent predictor of postoperative pain. These findings are largely consistent with the existing literature. Some studies [28, 29] have reported changes in vital signs during dental procedures; however, these changes were not found to be directly associated with the level of dental anxiety. In another study, higher anxiety, younger age, and a history of traumatic dental experiences were associated with greater increases in heart rate during the administration of local dental anesthesia [30]. Likewise, Özmen et al. [31] reported variations in vital signs among individuals with high dental anxiety. Such physiological alterations are thought to be related not only to stress induced by pain, fear, and anxiety during dental procedures, but also to individual characteristics including age, hypertension, history of traumatic dental treatment, psychological response patterns, unhealthy dietary habits, sedentary lifestyle, body mass index, and tobacco use [32]. In another study conducted in the Iranian population, a correlation was reported between dental anxiety, pain perception, and psychological factors [33]. In the present study, the absence of significant differences in vital signs among the anxiety groups supports the notion that the clinical effects of anxiety may not arise directly through hemodynamic or physiological changes, but rather through subjective and central mechanisms such as pain perception.

The results of the present study showed that a significant association between postoperative pain and sex was observed only on postoperative day 3 (*p* < 0.05), while no significant differences were detected during the remaining days of the 7-day follow-up period (*p* > 0.05). However, this finding was limited to a single time point and should be interpreted with caution. It may represent an incidental observation rather than a consistent pattern, and further studies are needed to confirm this finding. Previous studies have reported conflicting findings regarding the role of sex in postoperative pain. While some authors have indicated that women may experience or report higher levels of postoperative pain [34], others have suggested that the effect of sex on pain is minimal or inconsistent [18]. Another study [35] reported that women had higher pain expectations than men; however, the pain they experienced did not differ significantly from that of men. This heterogeneity may suggests that pain perception is shaped not only by biological factors but also by individual characteristics such as psychological state, pain expectations, and coping strategies. In the present study, the fact that the sex-related difference emerged only in the intermediate period and was transient supports the dynamic and multidimensional nature of postoperative pain. This finding suggests that sex is not a sole determining factor, but rather a variable that may become influential under certain conditions and during specific time periods.

When postoperative pain change (delta pain) was evaluated according to the anxiety groups in this study, statistically significant differences were observed among the groups only during the first three postoperative days, whereas these differences disappeared in the subsequent days. Preoperative pain is an important factor influencing postoperative pain perception. Although no significant differences were observed between groups at baseline, its potential confounding effect cannot be entirely excluded. Although delta pain analysis was used to explore temporal changes in pain, it does not replace adjusted modeling approaches. Therefore, multivariable regression analysis including preoperative pain was performed to more appropriately account for baseline differences. It can be proposed that the changes in pain observed in the initial days are more strongly influenced by psychological factors such as patients’ pain expectations, attentional focus, and emotional responses, whereas over time, the healing process and tissue repair become predominant, thereby attenuating these effects. The observation that delta pain differed significantly only within the first three postoperative days in the present study further indicates that the impact of anxiety on pain perception is temporary and varies over time rather than being sustained.

According to the results of the present study, analgesic consumption was higher in the groups with elevated anxiety levels. This finding may provide strong evidence that perceived pain was more severe in these patients. Although analgesic consumption differed significantly between groups, its potential confounding effect was further evaluated in an adjusted regression model. Analgesic use was not found to be a significant predictor of postoperative pain. This suggests that the influence of anxiety is not substantially explained by differences in analgesic use. Interestingly, despite higher analgesic consumption in the high anxiety group, these patients reported greater postoperative pain levels. This observation may suggest that the influence of anxiety on pain perception persists even in the presence of analgesic use, indicating a potential “masking effect.” However, this interpretation should be approached with caution, as analgesic use was not standardized and was recorded as a binary variable. These findings are consistent with previous studies, such as that of Qiao et al. [36], which demonstrated an association between higher dental anxiety and increased severity of postoperative symptoms, and is also consistent with the findings of Baagil [37], who reported that preoperative anxiety increases the need for postoperative analgesics. Likewise, Fernandez et al. [38] reported that patients with higher DAS scores used ibuprofen more frequently. From a clinical perspective, these findings suggest that postoperative pain control may be more challenging in patients with higher levels of anxiety. Moreover, analgesic consumption may represent a consequence of pain rather than a true independent determinant. However, in the present study analgesic use was recorded as a binary variable without detailed information on type, dosage, frequency, or duration, which limits the interpretability of this variable and its potential effect on postoperative pain. Analgesic consumption may have acted as an uncontrolled co-intervention that could have influenced postoperative pain outcomes.

Although previous studies have demonstrated an association between dental anxiety and pain, evidence specifically addressing postoperative pain following endodontic treatment remains limited. The present study builds upon existing literature by focusing on symptomatic vital mandibular molars and providing a prospective evaluation of postoperative pain over a seven-day period, along with relevant clinical and physiological parameters. The prospective design of the study allowed data to be collected in a forward-looking manner, thereby minimizing potential recall bias for patients. In addition, the evaluation of a seven-day postoperative period and the relatively large sample size constitute important strengths of the study. Monitoring postoperative pain for seven days provided an opportunity to assess the temporal variation in the influence of anxiety on pain outcomes. This longitudinal design revealed that anxiety-related differences were particularly evident in the early postoperative phase and decreased over time, offering a more detailed understanding of postoperative pain dynamics. Moreover, since the operator was unaware of the patients’ anxiety levels during treatment, the risk of operator-related bias was minimized. Furthermore, the study was not limited solely to postoperative pain; multiple factors, including anxiety level, sex, vital signs and analgesic consumption were evaluated simultaneously. The use of the MDAS, a well-validated instrument for assessing dental anxiety, provides a standardized approach to measuring anxiety levels. However, it should be noted that MDAS assesses general dental anxiety, therefore, the findings of this study should be interpreted as reflecting the influence of overall dental anxiety on postoperative pain, rather than anxiety specific to endodontic procedures. Furthermore, the lack of significant differences among the anxiety groups in terms of demographic variables, vital parameters, preoperative pain, and preoperative percussion pain confirms baseline comparability between the groups. This comparability suggests that the postoperative differences are more likely attributable to anxiety levels rather than baseline clinical or physiological factors, enhancing the internal validity of the study. Finally, evaluating analgesic intake as a separate clinical outcome variable enabled the assessment to focus not only on subjective pain scores but also on an objective clinical behavior. Demonstrating a significant association between anxiety level and analgesic use strengthens the clinical relevance of the study and supports the notion that anxiety influences the postoperative pain experience in a multidimensional manner.

Nevertheless, it should be acknowledged that the study has certain limitations. The single-center design of the study limits the generalizability of the findings to different populations. Although a reliable instrument such as the MDAS was used, the scale assesses only one dimension of anxiety, and no additional psychometric evaluations were performed. Moreover, since both pain assessment and analgesic use were based on patient self-report, recall bias and subjective variations in reporting may have occurred. Analgesic use was recorded as a binary variable without standardization or detailed information on type, dosage, frequency or timing. In addition, the exact timing of analgesic intake relative to pain assessment could not be controlled. These limitations restrict the ability to accurately evaluate the influence of analgesic consumption on postoperative pain outcomes. Variability in analgesic regimens among participants may have influenced pain perception and reporting, and therefore, findings related to analgesic use should be interpreted with caution.

Although the clinical protocol was standardized, specific procedural factors such as sealer extrusion, apical patency, or minor over-instrumentation were not systematically recorded. These factors may influence postoperative pain and represent a limitation of the present study. In the present study, intraoperative pain was not recorded as a separate variable. Although efforts were made to achieve adequate anesthesia in all cases, variations in intraoperative pain experience may have influenced patient-reported outcomes and represent a potential confounding factor.

Postoperative pain and delta pain analyses were limited to a seven-day follow-up period. Although this duration was sufficient to evaluate early and intermediate postoperative pain dynamics, it did not allow for the evaluation of long-term pain outcomes or potential late postoperative effects. Postoperative pain was analyzed at each time point separately rather than using a repeated measures framework. We acknowledge that this represents a methodological limitation, as this approach does not fully account for within-subject correlations and may increase the risk of type I error. Although a multivariable regression model was applied to adjust for potential confounders, the analytical approach did not fully account for the longitudinal structure of the data, which should be considered when interpreting the findings. Consequently, the findings should be interpreted with caution, and future studies employing longitudinal or mixed-effects models are warranted.

In future studies, to elucidate the effect of preoperative anxiety on postoperative pain more comprehensively, it is recommended that anxiety levels be assessed not only before treatment but also repeatedly throughout the treatment process and during the postoperative period. This approach may contribute to a clearer understanding of the time-dependent dynamics of the anxiety–pain relationship. In patient groups with high anxiety, investigating the use of behavioral techniques or pharmacological support before treatment and the individualization of postoperative analgesic therapy may contribute to clinical outcomes. In the present study objective physiological or biological stress markers, such as salivary cortisol levels, were not evaluated. The absence of objective stress markers may limit a more comprehensive understanding of the relationship between psychological and physiological aspects of anxiety. The inclusion of such measures may provide complementary data and should be considered in future studies. Conducting multicenter studies with larger sample sizes would enhance the generalizability of the findings and contribute to validating the relationship between anxiety and postoperative pain across different clinical settings and populations. Furthermore more detailed diagnostic classification according to standardized criteria and stratified analyses based on baseline pain severity may provide additional clinical insight and should be considered in future studies. Lastly, including more detailed information on analgesic use, such as type, dosage and frequency, may help to better understand its potential impact on postoperative pain.

## Conclusion

The present study demonstrates that postoperative pain experience following endodontic treatment in symptomatic molar teeth is influenced by preoperative dental anxiety levels, particularly during the early postoperative period. Patients with higher anxiety levels exhibited more pronounced pain and pain changes in the initial days, with this effect diminishing over time. In addition, the greater use of analgesics in the high-anxiety group supports the clinical relevance of these findings. Overall, the results suggest that assessing anxiety levels and implementing anxiety management strategies prior to endodontic treatment may improve pain control in the early postoperative period.

## Supplementary Information

Supplementary Material 1.

## Data Availability

The datasets used and/or analysed during the current study are available from the corresponding author on reasonable request.
