# Supplementary material for: The association between anxiety levels and postoperative pain following endodontic treatment: a prospective observational study
Source: BMC Oral Health. 2026 May 27;26:1445. doi: 10.1186/s12903-026-08715-7 (PMC13474775; doi:10.1186/s12903-026-08715-7)
Supplement: Supplementary file 1 [file 12903_2026_8715_MOESM1_ESM.pdf]

Can you tell us how anxious you feel during your dentist visit?
Please indicate by placing an 'X' in the appropriate box.

1.How would you feel if you were to go to your dentist for treatment tomorrow?

□ not anxious □slightly anxious □fairly anxious □very anxious □ extremely anxious

2. How would you feel if you were sitting in the waiting room (waiting for treatment)?

□ not anxious □slightly anxious □fairly anxious □very anxious □ extremely anxious

3. You sit in the dentist's chair and wait for your doctor to prepare the drilling instruments for treatment. How would you feel?

□ not anxious □slightly anxious □fairly anxious □very anxious □ extremely anxious

4. You sit in the dentist's chair and wait for your doctor to prepare the scaling tools to clean the tartar around your gums. How would you feel?

□ not anxious □slightly anxious □fairly anxious □very anxious □ extremely anxious

5. How would you feel if your dentist were about to local anesthesia injection into your tooth in the back area of the upper jaw?

□ not anxious □slightly anxious □fairly anxious □very anxious □ extremely anxious
